# Use of wearable technology in improving emergency care and health outcomes for patients with urgent health complaints: protocol for a scoping review

**DOI:** 10.1136/bmjopen-2025-106396

**Published:** 2026-03-03

**Authors:** Rawan Alotaibi, Alanowd Alghaith, Lukas Hughes Noehrer, Gareth B Kitchen, Richard Body

**Affiliations:** 1 Division of Cardiovascular Sciences, School of Medical Sciences, The University of Manchester, Manchester, UK; 2 Department of Emergency Medical Services, King Saud bin Abdulaziz University for Health Sciences College of Applied Medical Sciences, Alahsa, Saudi Arabia; 3 King Abdullah International Medical Research Centre, Alahsa, Saudi Arabia; 4 Social Science Applied Healthcare and Improvement Research (SAPPHIRE) Group, Department of Population Health Sciences, College of Life Sciences, University of Leicester, Leicester, UK; 5 Department of Emergency Medical Services, College of Applied Medical Science, King Saud bin Abdulaziz University for Health Sciences, Riyadh, Saudi Arabia; 6 King Abdullah International Medical Research Centre, Riyadh, Saudi Arabia; 7 Department of Anaesthesia, Critical Care and Perioperative Medicine, Manchester University NHS Foundation Trust, Manchester, UK; 8 Department of Computer Science, The University of Manchester, Manchester, UK; 9 Division of Immunology, Immunity to Infection and Respiratory Medicine, School of Biological Sciences, Faculty of Biology, Medicine and Health, The University of Manchester, Manchester, UK; 10 Emergency Department, Manchester University NHS Foundation Trust, Manchester, UK; 11 Division of Cardiovascular Sciences, The University of Manchester, Manchester, UK

**Keywords:** triage, wearable devices, accident & emergency medicine, emergency service, hospital

## Abstract

**Abstract:**

**Introduction:**

Since the 1970s, telemedicine has transformed significantly, becoming a critical component of modern healthcare delivery. Over time, technological innovation has increasingly emphasised the integration of the human body with digital systems to develop non-invasive methods for monitoring physiological parameters. Among these technologies, wearable sensors demonstrate substantial potential for continuous patient monitoring. These devices can facilitate real-time data collection, enable more rapid clinical decision-making and promote active patient participation in health management. Such capabilities are particularly valuable in emergency contexts, including prehospital care provided by ambulance services and telephone triage systems. Despite the growing interest in wearable health technologies, their integration into emergency medical services (EMS) remains insufficiently explored and warrants further investigation. We aim to map current research, explore the use of wearables in EMS settings and identify gaps in knowledge regarding their use in EMS.

**Methods and analysis:**

This scoping review will follow the Joanna Briggs Institute’s (JBI) methodology for scoping reviews. A systematic search of relevant databases (MEDLINE, EMBASE, Cochrane Library, CINAHL, ProQuest and Web of Science) will be conducted, from inception to March 2026. All types of study designs, including quantitative and qualitative studies, will be considered in this scoping review. The inclusion is limited to studies published in English. Two independent reviewers (RA and AA) will conduct a thorough screening of titles and abstracts against the predefined inclusion criteria. Studies that meet the inclusion criteria will be reviewed in full text. Quality and risk of bias will be assessed using the JBI’s critical appraisal tools for the relevant study types. The findings will be presented using diagrams or tables, supplemented by narrative summaries following the JBI guidelines.

**Ethics and dissemination:**

Ethical approval is not required. The findings of this study will be disseminated via publication in a peer-reviewed journal.

**Registration:**

Open Science Framework (10.17605/OSF.IO/MUEFX).

**STRENGTHS AND LIMITATIONS OF THIS STUDY**

- This scoping review will provide an overview of the evidence base on the use of wearables in emergency medical services settings, including types of wearables used, outcome measures, benefits and challenges and methods related to this topic.
- The review is designed to outline the use of wearables to improve patient outcomes and to identify the gaps in current research, in order to highlight areas where further research is needed.
- The review will be limited to publications available in the English language.

## Introduction

 Since the 1970s, telemedicine has transformed significantly, becoming a critical component of modern healthcare delivery. It was first mentioned in cardiology when electrocardiographic data were transmitted over telephonic wires.^1^ Since then, there has been a significant revolution in technology, focusing on the integration of the body and technology to find new, non-invasive ways to monitor physiological functions. There is now a growing industry of wearable technologies that are cost-effective and easy to use. The wearable market was valued at US$ 109.34 billion in 2023, and it is expected to exceed US$ 303.98 billion by 2029.^2^ Wearable technology, also known as wearables, biosensors or devices, typically refers to biological sensors including clothing (such as socks, blankets or vests, etc), watches, rings and others for continuous use. Wearables can be defined as small sensors that can be worn on the body and provide physical information to the user. Wearable devices are mainly divided into two categories: consumer and medical-grade devices. Examples include consumer products marketed as activity trackers from Fitbit^3^ or wellness wearables, such as smartwatches produced by Samsung or Apple.^4 5^ The wireless vital signs monitor, Radius VSM and Cosano Watch are considered as medical-grade devices to monitor the patients continuously.^6^,^8^ The emerging field of wearable sensors offers many capabilities in the medical field to monitor patients. These advantages include continuous monitoring, the possibility of a quick diagnosis and increased patient involvement in their health, which can improve care in emergency situations, especially in ambulance service/emergency medical services (EMS) or even through telephone triage. EMS refers to an organised system responsible for providing emergency care and transport to the injured and sick individuals to hospitals. This includes the evaluation, observation and treatment of patients by qualified healthcare providers such as emergency medical technicians and paramedics, starting from the moment of initial medical contact until the patient reaches a hospital.

Vital signs are an important part of the medical decision-making process because they allow healthcare professionals to make well-informed decisions.^9^ However, during mass casualty incidents or disasters, EMS resources are often limited, complicating timely assessment and intervention.^10^ The challenges of monitoring vital signs in the prehospital/out of hospital setting further complicate medical decision-making. Medical monitor mobility is a key component in field medicine and ambulatory care.^11^ Physiological trends can be more critical than single readings, as they allow paramedics to track changes more efficiently. Therefore, technologies that enable more regular, earlier and accurate measurements of vital signs are important for effectively triaging and treating the patients in early stages.

However, the true impact of wearable-derived vital signs lies not only in their measurement but in how promptly and effectively these data are communicated to emergency services. Call handlers are often the first link in the emergency response chain, making critical triage decisions based on limited information. In the UK, the ambulance service call handler uses the NHS 999 Pathways. The NHS 999 Pathways uses pre-triage questions to recognise life-threatening conditions by identifying them against predefined chief complaint and dispatching an ambulance. The pathway categorises the call into one of four categories. Category 1 requires an immediate response, including life-threatening conditions such as cardiac arrest, stroke and choking. Burns, serious injuries and sepsis are considered as category 2 with response time within 18 min. For an urgent but not a life-threatening condition, the call handler will ask the caller’s more questions to identify the caller’s condition. For non-urgent conditions, the call will be categorised as category 4.^12^ However, the response time of the ambulance is not as targeted. In March 2025, in England, 90% of patients of category 2 received a response in 58 min.^13^

Incorporating the data obtained from wearables into emergency call systems can improve the accuracy and speed of triage, empowering call handlers to prioritise calls based on both objective physiological indicators and patient symptoms rather than only subjective reports. Both physiological data and patient history can enable early identification of life-threatening conditions like acute coronary syndrome or severe trauma, encouraging timely dispatch of an ambulance. The use of real-time biometric data reduces the potential for under-triage, which can delay the ambulance response, thus delaying critical interventions, and over-triage, which can consume emergency resources. Moreover, the availability of physiological data at the call handling phase enables more accurate communication to paramedics en route, allowing for better preparation and focused intervention on arrival. It is reasonable to suggest that such a combination could support the enhancement of EMS by reducing response times, improving call prioritisation and enhancing overall system responsiveness. Despite the potential value of wearables in EMS, there is not enough research examining the incorporation of these devices into current care. Several reviews have explored wearable biosensors monitoring technologies in healthcare, particularly their use in chronic disease management, fitness tracking and hospital-based monitoring. However, these reviews have largely focused on technological performance, algorithm development or clinical validation in controlled environments.^14^,^16^ The literature does not sufficiently address the integration of real-time physiological data from wearables into emergency triage systems or communication pathways, such as those used by call handlers and ambulance services. The potential role of wearables in improving triage accuracy, response prioritisation and prehospital decision-making remains underexplored.

The majority of studies tend to concentrate on technologies while overlooking their combined effects on monitoring, triage systems and communication pathways, such as those used by call handlers and ambulance services. Therefore, this scoping review is needed to synthesise existing evidence on the use of wearable technology within EMS settings, identifying current applications, challenges and research gaps related to their integration into emergency care workflows.

### Aim and objective

This scoping review aims to explore and map the existing literature on wearable technology applications in EMS, focusing on their impact on emergency care and improving health outcomes for patients with health concerns. The specific study objectives are to identify the benefits and challenges associated with the use of wearable technologies to improve EMS triage, examine how studies characterised the effectiveness of wearable technology on patient outcomes and identify gaps in the current body of knowledge regarding the integration of wearable technology into emergency systems.

### Research question

How can wearable technology be used to enhance emergency care and improve health outcomes for patients with health complaints in EMS settings?

## Methods and analysis

### Scoping review design

**Figure 1:**
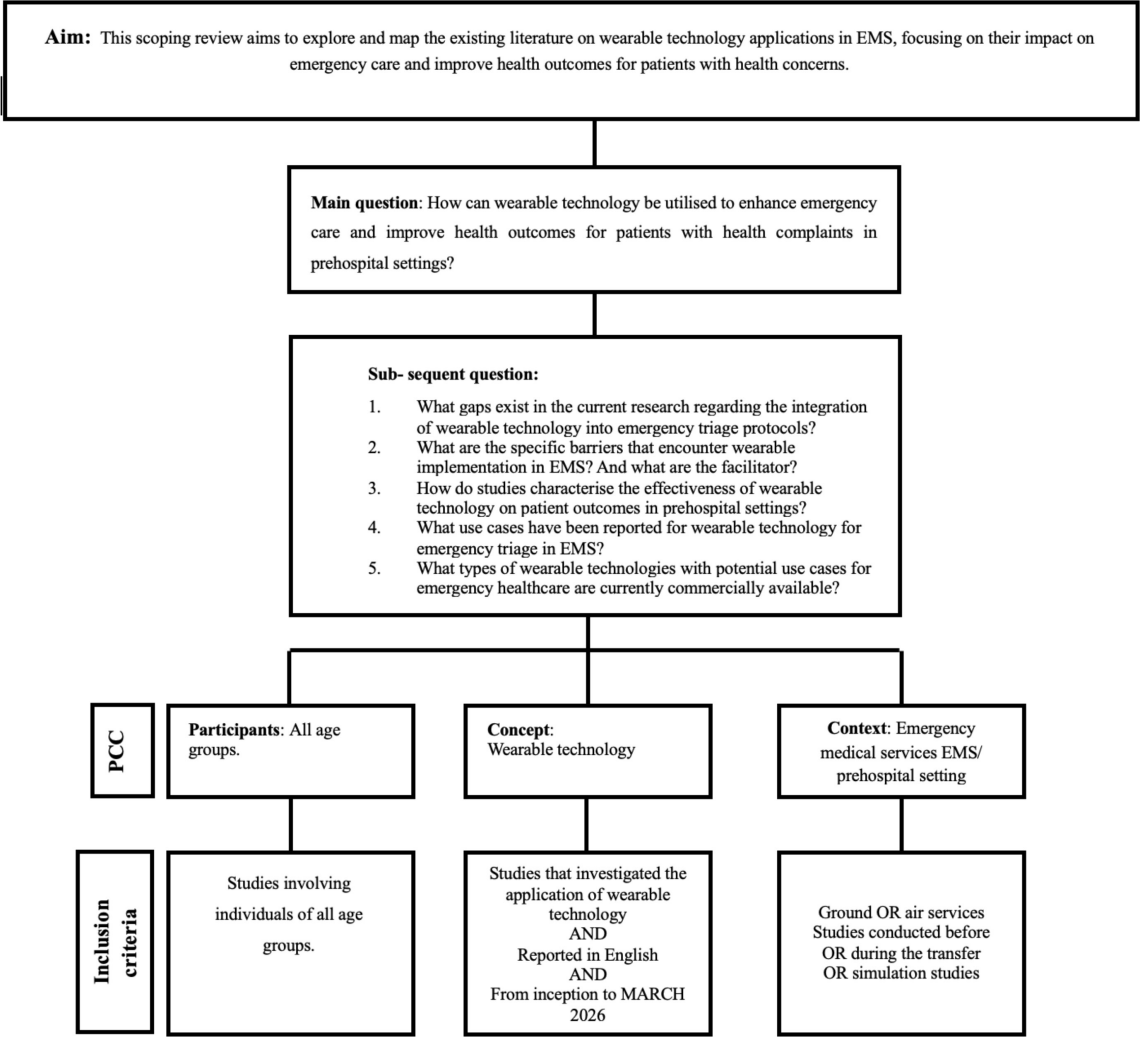
Participants, concept, context framework, reflecting the research aim, questions and eligibility criteria. EMS, emergency medical services; PCC, population/participants, concept, context.

**Figure 2:**
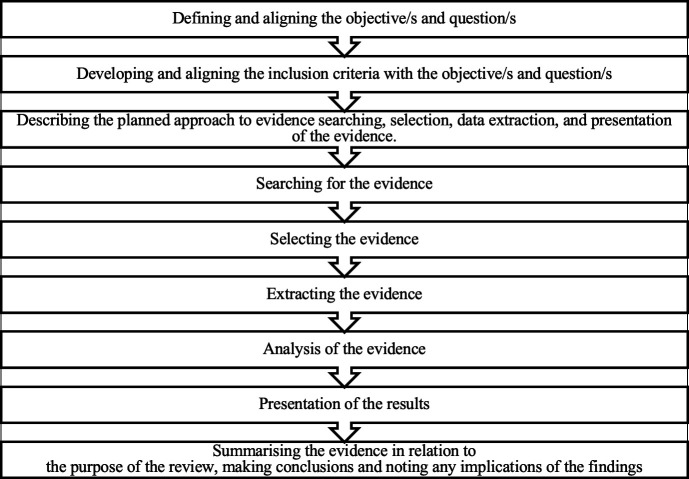
Joanna Briggs Institute nine-stage framework for the scoping review.

The planned scoping review will follow the Joanna Briggs Institute (JBI) methodology and enhancements^17^ to map and summarise the literature on wearable technology in emergency care and to identify research gaps. The review will use the population/participants, concept, context framework presented in figure 1. The review will be organised into nine stages (figure 2), ensuring a comprehensive exploration of the benefit of implementing wearable technology on patient outcome.

### Review registration

This review protocol has been registered with the Open Science Framework.^18^

### Review team

This review team is composed of multidisciplinary expert clinicians and academics in the field of emergency medicine (RB), anaesthesia (GBK), prehospital care (RA and AA) and computational medicine (LHN).

### Eligibility criteria

#### Participants

The scoping review aims to include studies involving individuals of all age groups. We did not specify age groups as we aim to understand how wearable technologies are used, their outcomes in emergency scenarios which are not age dependent. This study includes all patients who require emergency medical assistance, focusing on patients who call for emergency help with a health concern (999, 112, 911, etc). A health concern is any condition that impacts an individual in a negative way which can be physical, mental, and from diseases and injuries, environmental influences and access to healthcare.

#### Concept

The scoping review will include studies that investigate the application of wearable technology. Additionally, the review will concentrate on the features of wearable technology such as their capacity to track vital signs (eg, heart rate, oxygen saturation and temperature) instantly. Furthermore, the review will consider studies that investigate how wearable technology can enhance the accuracy and efficiency of triage decisions, improve patient outcomes or streamline the communication between patients and healthcare professionals in emergency contexts. Consequently, studies addressing these key concepts and functionalities will be included in the review.

#### Context

The scoping review will focus on studies that investigated wearables in the EMS context, referring to organised out of hospital/prehospital systems that provide emergency medical assessment including ground and air ambulance, while excluding studies carried out in primary care facilities, hospital emergency departments, intensive care units, hospital wards or military scenarios.

#### Types of studies

Qualitative, quantitative and mixed-methods studies, as well as grey literature (eg, conference abstract).

### Search strategy

The search strategy of the scoping review is designed to be systematic and comprehensive and is comprised of three stages. The initial stage will be performing an electronic search in Medline and EMBASE (Ovid) databases to identify keywords (table 1). Then, based on the keywords identified in the initial stage, the second stage focuses on a more structured list of keywords to identify the most relevant studies pertaining to the research question with the assistance of an academic librarian. To validate the selected keywords, the opinions and recommendations of two field experts will be sought to guarantee that the search strategy encompassed a broad spectrum of terms related to the research question. This search term will be used in six major databases: MEDLINE, EMBASE, Cochrane Library, CINAHL, ProQuest and Web of Science from inception to March 2026.

**Table 1:** Initial search keywords

Participants	N/A
Concept	Smartwatch, wearable
Context	Emergency medical service, paramedic, ambulance

The review will also explore grey literature and unpublished studies, such as reports and conference abstracts, to ensure a comprehensive scope. All types of study designs, both qualitative and quantitative, will be considered. Finally, to collect research that fits the inclusion criteria of the reference lists, the included studies will be examined using the ‘related articles’ feature. Before finalising the search strategy, we conducted a pilot screening in June 2025 to ensure it would yield relevant and sufficient results (online supplemental table 1).

### Study/source of evidence selection

In order to capture a more comprehensive and wide body of evidence, no language restriction will be applied in the selection of studies process. After conducting the database search, the results will be uploaded to Rayyan software to eliminate duplicates and streamline the organisation and tracking of the screening process.^19^ Then, the selection process will follow a two-step screening approach: first, all identified studies will undergo initial screening based on titles and abstracts by two independent reviewers (RA and AA). Second, studies that meet the inclusion criteria will be reviewed in full text, and eligibility criteria are presented in online supplemental table 2. In the case of disagreements between the two reviewers, resolution will be achieved through discussion or consultation with a third senior reviewer.

The study inclusion process and the search results will be comprehensively reported in the final scoping review by a preferred reporting items for systematic reviews and meta-analyses extension for scoping review flow diagram to present a structured overview of the selection process.^20^

### Data extraction

The data will be extracted using a predeveloped standardised data extraction form, which will capture study characteristics (outlined in online supplemental table 3). This will be piloted by two researchers before finalising the content, and any changes made will be documented in the full review paper.

### Critical appraisal of individual sources of evidence

The JBI’s critical appraisal tool will be used to conduct a critical appraisal of the included studies.^21^ This quality assessment aims to evaluate the methodological rigour and reliability of the existing literature.

### Presentation of the results

Descriptive and narrative synthesis will be used to summarise the findings from the included studies. Temporal and geographical trends in publication of relevant material will be summarised by creating tables and charts, using descriptive data (eg, number of publications by year, stratified by study type).

The nature of individual study reports will be presented through tables that summarise the general characteristics of each study. Key demographics and outcome variables from the included publications will be summarised using descriptive statistics. The studies will be classified according to their target population and type of wearable technology.

The extracted information will be categorised based on the research questions, which cover various aspects:

1. The utilisation of wearable technology to enhance emergency care and improve health outcomes for patients with health complaints in EMS.
2. The benefits and challenges associated with the use of wearable technology in emergency triage.
3. The impact of wearable technology on health outcomes for patients with health concerns.

### Patient and public involvement

None.

## Ethics and dissemination

As this study is a scoping review of publicly available literature, ethical approval is not required. The study was reviewed using the University of Manchester Ethics Decision Tool and classified as ethically exempt in accordance with university policy. The findings of this study will be disseminated by publication in a peer-reviewed journal, analyse results to determine any areas for full systematic review, and to identify key gaps in the literature for future work.

## Supplementary material

10.1136/bmjopen-2025-106396 online supplemental file 1

## References

[1] Raikhelkar J, Raikhelkar JK (2015). The impact of telemedicine in cardiac critical care. Crit Care Clin.

[2] Smart Wearable Market (2025). Global outlook & forecast 2024-2029. https://www.researchandmarkets.com/report/smart-wearable?utm_source=GNE&utm_medium=PressRelease&utm_code=qtm5kq&utm_campaign=2050839+-+Smart+Wearable+Global+Market+Report+2025%3a+Smartwatches%2c+Earwear+and+Health+Rings+are+Major+Growth+Segments+-+Global+Smart+Wearable+Market+to+Surpass+%24300+Billion+by+2029&utm_exec=carimspi.

[3] (2025). Fitbit homepage. https://store.google.com/ie/category/watches_trackers?hl=en-GB&utm_source=fitbit_redirect&utm_medium=google_ooo&utm_campaign=category.

[4] (2025). Samsung. https://www.samsung.com/uk/watches/?product1=sm-l705fdaaeua&product2=sm-l305fzgaeua&product3=sm-r861nzsaeua.

[5] (2025). Apple watch. https://www.apple.com/uk/watch/.

[6] Meizoso JP, Allen CJ, Ray JJ (2016). Evaluation of Miniature Wireless Vital Signs Monitor in a Trauma Intensive Care Unit. Mil Med.

[7] Wire WB Masimo announces limited market release of radius VSM.

[8] Continuous patient monitoring. https://corsano.com.

[9] ATLS Advanced trauma life support. https://www.facs.org/quality-programs/trauma/education/advanced-trauma-life-support/?page=1.

[10] McGuire NM (2006). Monitoring in the field. Br J Anaesth.

[11] Sward DG, Bennett BL (2014). Wilderness medicine. World J Emerg Med.

[12] Turner RJ, Crum A, Coster J (2017). Ambulance response programme.

[13] NHS (2025). Ambulance quality indicators. https://www.england.nhs.uk/statistics/wp-content/uploads/sites/2/2025/04/20250410-Note-Statistical-AQI-1.pdf.

[14] Patel V, Orchanian-Cheff A, Wu R (2021). Evaluating the Validity and Utility of Wearable Technology for Continuously Monitoring Patients in a Hospital Setting: Systematic Review. JMIR Mhealth Uhealth.

[15] Mattison G, Canfell O, Forrester D (2022). The Influence of Wearables on Health Care Outcomes in Chronic Disease: Systematic Review. J Med Internet Res.

[16] Lodewyk K, Wiebe M, Dennett L (2025). Wearables research for continuous monitoring of patient outcomes: A scoping review. *PLOS Digit Health*.

[17] Aromataris ELC, Porritt K, Pilla B (2024). JBI manual for evidence synthesis.

[18] Alotaibi RS, Body R, Alghaith A (2025). The use of wearable technology in improving emergency care and improve health outcomes for patients with urgent health concerns in prehospital setting: a scoping review protocol. https://osf.io/muefx/overview.

[19] Ouzzani M (2016). Rayyan — a web and mobile app for systematic reviews. https://www.rayyan.ai.

[20] Tricco AC, Lillie E, Zarin W (2018). PRISMA Extension for Scoping Reviews (PRISMA-ScR): Checklist and Explanation. Ann Intern Med.

[21] (2020). Critical appraisal tools. https://jbi.global/critical-appraisal-tools.

