# Supplementary material for: Use of wearable technology in improving emergency care and health outcomes for patients with urgent health complaints: protocol for a scoping review
Source: BMJ Open. 2026 Mar 3;16(3):e106396. doi: 10.1136/bmjopen-2025-106396 (PMC12958921; doi:10.1136/bmjopen-2025-106396)
Supplement: online supplemental file 1 [file bmjopen-16-3-s001.docx]

**Supplementary Table 1.** Pilot search result (June 2025)

Embase <1980 to 2025 Week 25>

| **#** | **Query** | **Results from 23 Jun 2025** |
| --- | --- | --- |
| 1 | exp wearable device/ | 22,691 |
| 2 | exp smart watch/ | 7,410 |
| 3 | exp activity tracker/ | 6,194 |
| 4 | [wearable.mp](http://wearable.mp/). | 40,827 |
| 5 | smart?watch$.mp. | 2,019 |
| 6 | fitness tracker$.mp. | 684 |
| 7 | exp emergency health service/ | 370,149 |
| 8 | exp ambulance/ | 18,763 |
| 9 | exp paramedical personnel/ | 644,695 |
| 10 | (emergency medical service$ or paramedic$ or ambulance$ or EMS or "999" or "911" or "112").mp. | 390,148 |
| 11 | 1 or 2 or 3 or 4 or 5 or 6 | 50,165 |
| 12 | 7 or 8 or 9 or 10 | 1,317,709 |
| 13 | 11 and 12 | 2,112 |

## **Supplementary Table 2.** Study eligibility

| Study Characteristics | Eligibility criteria | | Eligibility criteria met. | | | Location in text or source *.* |
| --- | --- | --- | --- | --- | --- | --- |
|  |  |  | Yes | No | Unclear |  |
| Participants | - Studies involving human - All age - Health Concerns | |  |  |  |  |
| Concept | The study investigating the application of wearable technology | |  |  |  |  |
| Context | The study investigating n wearables in prehospital settings | |  |  |  |  |
| INCLUDE | | EXCLUDE | | | | |
| Reason for exclusion |  | | | | | |
| Notes: | | | | | | |

|  |  |
| --- | --- |
|  |  |
|  |  |

## **Supplementary Table 3.** Data extraction form

| Study information | Title | |  |
| --- | --- | --- | --- |
|  | Author | |  |
|  | year | |  |
|  | country | |  |
|  | Primary objective | |  |
|  | Secondary objective | |  |
| Methodology | Start date | |  |
|  | End date | |  |
|  | Study design | |  |
|  | Type of wearable | |  |
|  | The manufacture | |  |
|  | Year of manufacture | |  |
|  | Sample size | |  |
| Results | Participants (Demographic information) | Age |  |
|  |  | Gender |  |
|  |  | Ethnicity |  |
|  | Primary outcome | |  |
|  | Secondary outcome | |  |
|  | Recommendations | |  |
|  | Conclusion | |  |
