# Supplementary material for: Use of wearable technology in improving emergency care and health outcomes for patients with urgent health complaints: protocol for a scoping review
Source: BMJ Open. 2026 Mar 3;16(3):e106396. doi: 10.1136/bmjopen-2025-106396 (PMC12958921; doi:10.1136/bmjopen-2025-106396)
Supplement: Supplementary file 2 [file bmjopen-2025-106396.reviewer_comments.pdf]

PEER REVIEW HISTORY

BMJ Open publishes all reviews undertaken for accepted manuscripts. Reviewers are asked to complete a checklist review form and are provided with free text boxes to elaborate on their assessment. These free text comments are reproduced below.

ARTICLE DETAILS

Title (Provisional)

The use of wearable technology in improving emergency care and health outcomes for patients with urgent health complaints: protocol for a scoping review

Authors

Alotaibi, Rawan; Alghaith, Alanowd; Noehrer, Lukas Hughes; Kitchen, Gareth B; Body, Richard

---

VERSION 1 - REVIEW

**Reviewer**	**1**
**Name**	**Dieckelmann, Mirjam**
**Affiliation**	**Goethe-Universitat Frankfurt am Main Institut fur Allgemeinmedizin**
**Date**	**18-Sep-2025**
**COI**	**None**

Thank you for the opportunity to review this scoping review protocol mapping the evidence around wearable technologies in pre-hospital emergency services. This study protocol complies with good methodological standards andwill be an interesting read for the journals readership. Some minor comments:

- The search strategy at this stage seems to be still very basic. It does not include filters, although there may be interesting paramedic filters published here https://pmc.ncbi.nlm.nih.gov/articles/PMC5637081/ or (non-validated) wearable filters published here, for example: https://www.sciencedirect.com/science/article/pii/S2352648325000169#mmc1 If filters will be applied, please reference them

- Do the authors have an estimate of how many publications they will identify? With the presented search strategy, that is very broad, it seems more realistic to apply tools for automated screening. If this is planned, please add which tools will be applied.

**Reviewer**	**2**
**Name**	**Tiase, Victoria**
**Affiliation**	**University of Utah, College of Nursing**
**Date**	**20-Sep-2025**
**COI**	**None**

Thank you for this interesting study protocol. I have a few minor recommendations to improve this work:

1. The one scoping question should be stated in the aims and objectives.

2. Please define how you plan to scope EMS for the purposes of the review. This could mean different things to different readers.

3. The gaps in the literature should be identified in the intro - why is this review needed? There are reviews in the sensor space that would be worthwhile to mention.

4. It would be helpful to have a table with inclusion and exclusion criteria. Are you including any country, any language?

5. Will a research librarian be supporting the search? If so, please include.

6. Measuring effectiveness was mentioned in the abstract. However, not seeing it in the abstraction tool. How will this be assessed?

7. In the summary, 'improving patient outcomes' is mentioned as well. I think you will need to pick one or the other or both and ensure this is aligned across the protocol.

8. Dates for this review are missing, please add.

VERSION 1 - AUTHOR RESPONSE

Reviewer: 1	The search strategy at this stage seems to be still very basic. It does not include filters, although there may be interesting paramedic filters published here https://pmc.ncbi.nlm.nih.gov/articles/PMC5637081/ [pmc.ncbi.nlm.nih.gov] or (non-validated) wearable filters published here, for example: https://www.sciencedirect.com/science/article/pii/S2352648325000169#mmc1 [sciencedirect.com] If filters will be applied, please reference them<br>Do the authors have an estimate of how many publications they will identify? With the presented search strategy, that is very broad, it seems more realistic to apply tools for automated screening. If this is planned, please add which tools will be applied.	1. Thank you for your feedback. We did initially try an alternative set of keywords for the search strategy; however, this yielded almost no results. As a result, we developed a broader strategy aimed at maximizing specificity without compromising sensitivity. To ensure that we were capturing relevant studies and refining our approach appropriately, we also conducted a pilot screening in Embase.<br>2. We will use Rayyan to help in screening process. Sample of the search result were added in the appendix

Reviewer: 2	The one scoping question should be stated in the aims and objectives.<br>Please define how you plan to scope EMS for the purposes of the review. This could mean different things to different readers.<br>The gaps in the literature should be identified in the intro - why is this review needed? There are reviews in the sensor space that would be worthwhile to mention.<br>It would be helpful to have a table with inclusion and exclusion criteria. Are you including any country, any language?<br>Will a research librarian be supporting the search? If so, please include.<br>Measuring effectiveness was mentioned in the abstract. However, not seeing it in the abstraction tool. How will this be assessed?<br>In the summary, 'improving patient outcomes' is mentioned as well. I think you will need to pick one or the other or both and ensure this is aligned across the protocol.<br>Dates for this review are missing, please add.	3. Done<br>4. Added in the introduction.<br>5. done<br>6. added<br>7. Yes, a research librarian is supporting the development of the search strategy, as mentioned in the relevant section.<br>8. change it to map the literature and see kind of wearables are used in EMS especially triage<br>9. I deleted the summery based on the first reviewer feedback.<br>10. Thank you for your comment. The initial search for this review will be conducted in June 2025. If necessary, the search will be updated prior to final analysis to ensure inclusion of the most recent and relevant studies.

VERSION 2 - REVIEW

Reviewer 1

Name Dieckelmann, Mirjam

Affiliation Goethe-Universitat Frankfurt am Main Institut fur Allgemeinmedizin

Date 05-Jan-2026

COI

A search strategy and estimated number of papers to be identified is now included in the appendix. This increases transparency. However, the authors now report that the search has been conducted before sumbission of the protocol (06/2025). Do I understand it correctly that these were pilot searches? If they weren't pilot searches, please state whether the protocol will be finalized before study selection and data charting.

Reviewer 2

Name Tiase, Victoria

Affiliation University of Utah, College of Nursing

Date 13-Jan-2026

COI

Thank you for addressing most of the reviewers' comments. I have a few outstanding recommendations to improve this manuscript:

1. Although there is a short sentence to define EMS, I still think this needs further clarification. Will urgent care centers be included? How about the technologies used by EMTs?

2. Connected to comment #1, a table with inclusion and exclusion criteria is very important. Your response indicates that this has been completed, but I don't see it. Please note which countries will be included and then include all possible emergency care setting definitions. I'm worried that this work is considering emergency care from a particular country health system lens. (which is fine, but if so, please indicate)

3. Prehospital is quite specific to the UK NHS. It may be helpful to include the corresponding terms from other countries if planning to search globally.

4. Dates of the search are confusing. There is the date that you conduct the search and then the data parameters for the search. Please adjust accordingly.

VERSION 2 - AUTHOR RESPONSE

Reviewer 1	1. A search strategy and estimated number of papers to be identified is now included in the appendix. This increases transparency. However, the authors now report that the search has been conducted before submission of the protocol (06/2025). Do I understand it correctly that these were pilot searches? If they weren't pilot searches, please state whether the protocol will be finalized before study selection and data charting.	1. Thank you for this comment. The searches conducted in June 2025 were **pilot searches**, undertaken to test and refine the search strategy, assess the feasibility of the review, and estimate the volume of potentially relevant literature.
Reviewer 2	2. Although there is a short sentence to define EMS, I still think this needs further clarification. Will urgent care centers be included? How about the technologies used by EMTs?<br>3. Connected to comment #1, a table with inclusion and exclusion criteria is very important. Your response indicates that this has been completed, but I don't see it. Please note which countries will be included and then include all possible emergency care setting definitions. I'm worried that this work is considering emergency care from a particular country health system lens. (which is fine, but if so, please indicate)<br>4. Prehospital is quite specific to the UK NHS. It may be helpful to include the corresponding terms from other countries if planning to search globally.<br>5. Dates of the search are confusing. There is the date that you conduct the search and then the data parameters for the search. Please adjust accordingly.	2. Thank you for this comment. In this review, Emergency Medical Services (EMS) are defined as organised out-of-hospital/prehospital emergency care systems providing emergency medical assessment by paramedic and EMT.<br>3. Thank you for this comment. The inclusion and exclusion criteria are presented in Figure 1, which outlines the eligibility criteria across participants, concept, context, and types of studies.<br>4. Thank you for this comment. We acknowledge that the term “prehospital” may be UK centric. To ensure clarity and support a global search, the protocol now includes corresponding terms used internationally, out-of-hospital care, emergency medical services.<br>5. Thank you for this comment. We have clarified the search

		dates in the manuscript. The pilot search was conducted in June 2025 to refine the strategy and estimate the number of relevant studies.
